# Genomics of Childhood Obesity

**DOI:** 10.2174/138920211795677949

**Published:** 2011-05

**Authors:** Merlin G Butler

Genomics of childhood obesity is an important and timely topic for investigators to better understand the role of gene function in the causation of childhood obesity, a significant health problem affecting much of westernized societies. Genetic abnormalities account for much of childhood obesity, syndromic and non-syndromic. Completion of the Human Genome Project and availability of genome sequencing data along with recent technical advances of chromosome microarrays, coding and non-coding RNA expression, high throughput testing platforms and next generation sequencing with bioinformatic tools has led to identification of genetic defects for early diagnosis of obesity-related syndromes impacting intervention, treatment and quality of life. 

The identification of gene mutations and characterization of gene alterations and subsequent protein disturbances in obesity are important in understanding causative genetic mechanisms and providing new insights into deciphering the complex networks of coding and non-coding gene expression that regulate obesogenic pathways. The role of epigenetics in this active field of research is also becoming more established.

Our current understanding of genetic factors contributing to childhood obesity will be addressed in this special journal issue with the aim to cover the latest developments in the genomics of childhood obesity by leading experts in their field. The first chapter is written by Drs. Choquet and Meyre as they review the molecular basis of obesity and the application of genomics. In their second review article, they summarize the current understanding of genetics and insight into childhood obesity with lessons learned through research. This article is followed by a report from Dr. Garver on diet and gene interaction pertaining to obesity in humans and the use of mouse models. Dr. Dasouki and colleagues report, in a separate review, their experience from a clinical standpoint, examples of individuals with obesity and structural chromosome anomalies with review of literature. The remaining three review articles focus on obesity-related genetic syndromes due to different mechanisms including Prader-Willi syndrome, the first example of errors in genomic imprinting in humans with gene expression dependent on the parent of origin, authored by myself; the fragile X syndrome due to a triplet repeat mutation with a subset of individuals with marked obesity is contributed by Dr. Hagerman and others; and Alström syndrome due to a mutation of a gene controlling ciliary function in cells contributing to obesity is written by Ms. Marshall and colleagues.

Although an attempt is made to provide the most accurate information regarding the genomics of childhood obesity and to provide examples of obesity-related genetic syndromes and their causation, this review is not meant to be exhaustive. Our aim is to have this special journal issue review the molecular basis of obesity and lessons learned through genetics research that impacts on the causation of childhood obesity, epigenetics and diet-gene interaction, clinical examples of obesity and structural chromosome abnormalities and description of several rare and uncommon genetic disorders with specific genetic lesions associated with obesity. This review should be useful to pediatricians, geneticists, endocrinologists and other health care providers engaged in the diagnosis, treatment and care of children presenting with obesity (syndromic and non-syndromic). The basic scientist engaged in research to discover and describe genetic mechanisms leading to the cause of obesity, particularly childhood, should benefit from reading this special journal issue on the genomics of childhood obesity. 

